# Perilesional sampling: the new standard for imaging-targeted prostate biopsies?

**DOI:** 10.1038/s41391-022-00634-2

**Published:** 2023-01-11

**Authors:** Christian Thomas

**Affiliations:** Department of Urology, Technische Universität Dresden, Dresden, Germany

**Keywords:** Outcomes research, Biomarkers

Magnetic resonance imaging-guided prostate biopsies have revolutionized the detection of clinically significant prostate cancer (csPCa) within the last decade [1]. Using a combined approach by targeted biopsies (TB) plus systemic biopsies (SB), the detection of csPCa significantly increases [2]. It is still under debate how SB add sensitivity to TB and might be even omitted in selected cases, but the risk of detecting up to 15% of csPCa outside the region of interest (ROI) favors the combined approach in current guidelines [1, 3].

The authors of the study presented here have done a well thought out analysis to determine the distance between systematic cores containing csPCa and the ROI in 505 consecutive patients undergoing TB plus SB [4]. This promising approach is based on the hypothesis that a so called “penumbra”, a radius around the ROI, bears a high likelihood of cancer detection and will be sufficient for additional biopsies outside the ROI. Noujeim et al. showed that perilesional sampling plus TB was non-inferior to SB plus TB in the detection fo csPCa (32% vs. 37%). The cumulative cancer distribution rate for csPCa reached 86% for the 10 mm margin. These data confirm the results of Brisbane et al., who demonstrated in a series of 2048 men undergoing MRGB plus SB that 90% of csPCa were located within an 10 mm radius from the ROI [5]. While Hansen et al. already showed in 2020 that a targeted saturation biopsy ipsilateral to the ROI is most effective for cancer diagnosis, the diameter of the “penumbra” in relation to the PIRADS-score of the ROI is a matter of debate [6].

In 2019, the PIRADS-committee recommend TB of the ROI and a 5-mm penumbra for PIRADS 4 and 5 lesions [7]. Moreover, Tafuri et al. found that for PIRADS 5 lesions with a PSA density of >0.15 ng/ml, systematic samples did not provide additional diagnostic yield [8]. Contrary, the presented study demonstrates that perilesional detection of csPCa not only depends on PIRADS-score but also on PSA density. Using a chi-square automated interaction detector (CHAID) machine learning algorithm and defining three risk groups by PIRADS-score and PSA density, the risk of missing csPCa beyond the 10 mm “penumbra” was 2%, 8%, and 29% for low-, intermediate- and high-risk groups, respectively. While in men with PIRADS 3–5 lesions and a PSA density <0.15 ng/ml biopsies outside the 10 mm “penumbra” can be omitted, standard TB plus SB is needed for PIRADS 4/5 lesions with a PSA density >0.15 ng/ml.

In conclusion, this study significantly contributes to the understanding of perilesional sampling in men undergoing prostate biopsy. However, confirmatory studies are needed to show if a risk-group based approach by PIRADS-score and PSA density has the potential to set up TB plus perilesional sampling biopsies as a new standard.
